# Context Modulation of Facial Emotion Perception Differed by Individual Difference

**DOI:** 10.1371/journal.pone.0032987

**Published:** 2012-03-14

**Authors:** Tae-Ho Lee, June-Seek Choi, Yang Seok Cho

**Affiliations:** 1 Department of Psychology, Korea University, Seoul, Korea; 2 Department of Psychology, University of Southern California, Los Angeles, California, United States of America; University of Bologna, Italy

## Abstract

**Background:**

Certain facial configurations are believed to be associated with distinct affective meanings (i.e. basic facial expressions), and such associations are common across cultures (i.e. universality of facial expressions). However, recently, many studies suggest that various types of contextual information, rather than facial configuration itself, are important factor for facial emotion perception.

**Methodology/Principal Findings:**

To examine systematically how contextual information influences individuals’ facial emotion perception, the present study estimated direct observers’ perceptual thresholds for detecting negative facial expressions via a forced-choice psychophysical procedure using faces embedded in various emotional contexts. We additionally measured the individual differences in affective information-processing tendency (BIS/BAS) as a possible factor that may determine the extent to which contextual information on facial emotion perception is used. It was found that contextual information influenced observers' perceptual thresholds for facial emotion. Importantly, individuals’ affective-information tendencies modulated the extent to which they incorporated context information into their facial emotion perceptions.

**Conclusions/Significance:**

The findings of this study suggest that facial emotion perception not only depends on facial configuration, but the context in which the face appears as well. This contextual influence appeared differently with individual’s characteristics of information processing. In summary, we conclude that individual character traits, as well as facial configuration and the context in which a face appears, need to be taken into consideration regarding facial emotional perception.

## Introduction

Imagine a poker player trying to guess an opponent’s hand. The player "reads" some ambiguous information about the opponent's hand in the opponent's facial expression. The perception of facial cues largely depends on the perceiver. The hand (e.g., busted hand or full house) and the character (e.g., nervous cat or bold lion) possessed by the player can greatly influence the player's interpretation of the opponent's subtle facial expressions. Despite general acceptance that context and individual factors influence people's abilities to perceive others' emotions via their expressions, most face perception studies have focused on the universality of the expressions. These studies have provided a strong theoretical foundation for a robust connection between a certain set of facial configurations and so-called “basic emotions"[1].

Recent studies have challenged this view [2]–[4]. Research has shown that various external surrounding factors, such as race [5], body posture [6], [7], verbal explanation of situation [8], [9], other emotional faces [10], and emotional scene [11]–[13] modulate the emotion perception of facial configuration. For example, in Aviezer et al.’s study (2008), which asked observers to rate the valence and arousal of a face embedded in varying contexts (e.g., body posture), observers recognized the same facial expressions as different emotions depending on the face's contextual image. These studies have shown that context information plays a role at an early, visual stage involved in face encoding processing [6], [14], at a late, processing stage involved in interpretation and perceptual decision [8], or at both the stages [12], [13], [15]. Together, these earlier results highlight the importance of surrounding circumstances, namely, context information, for perceiving emotions from facial configuration.

However, not all studies necessarily showed that context information is a predominant factor in facial emotion perception [16], [17]. For example, in Nakamura et al.’s study (1990), when participants rated the emotional state of a facial configuration presented simultaneously with situational information, facial configuration itself had a larger effect on the emotion rating than situational information. The authors concluded that the recognition of facial emotion is primarily determined by facial configuration.

It has been found that information processing of emotional stimuli varies as a function of individual differences (see [18] for a review). For example, Carver and White demonstrated that individual’s behavioral tendencies of approach and avoidance are closely related to the tendency to process emotional information [19]. Therefore, individual differences could have affected the extent to which observers used emotional context information in facial emotion perception in the previous studies. In light of this, the present study focuses on each individual’s Behavioral Inhibition System (BIS), which is conceptually linked to approach motivation, and Behavioral Approach System (BAS; [19], [20]), which is linked to avoidance motivation, because BIS/BAS levels are associated, respectively, with the processing of negative and positive information [21], [22]. In particular, both the BIS [23] and BAS [24] modulate the activity of the amygdala, a central component in emotion processing. Thus, the present study examines the influences of individual’s BIS/BAS on facial emotion perception with emotionally different contextual information.

The present study conducted a face-emotion perceptual task to test the roles of both context information and tendency to attend to emotional information in facial emotion perception. There are several ways to define the concept of context. For example, Chun (2000) defined it as elements and the way those elements are configured to form a complex set of stimuli [25]. However, in the present study, context is defined as a functionally relevant set of stimuli that can modulate a response, but not directly drive it, often by influencing how information is sampled from other stimuli that are more causally linked to the target response [26], [27]. For this task, a range of morphed faces, with varying emotional intensities, was overlaid on one of three emotional contexts: negative, positive, or neutral. Observers were asked to indicate whether the face in each contextual image was fearful or neutral and observers’ behavioral performance was characterized via psychometric curves. In this manner, we directly estimated each observer’s perceptual thresholds for identifying a face as fearful in different contexts. To assess the modulatory effect of individual differences, we measured the observers’ affective information-processing tendency regarding BIS/BAS.

## Methods

### Observers

The study was approved by the Ethics Committee of the Korea University. Fifteen observers with corrected-to-normal vision volunteered for this study. We obtained written informed consent from all observers.

### Stimuli and apparatus

**Figure 1:**
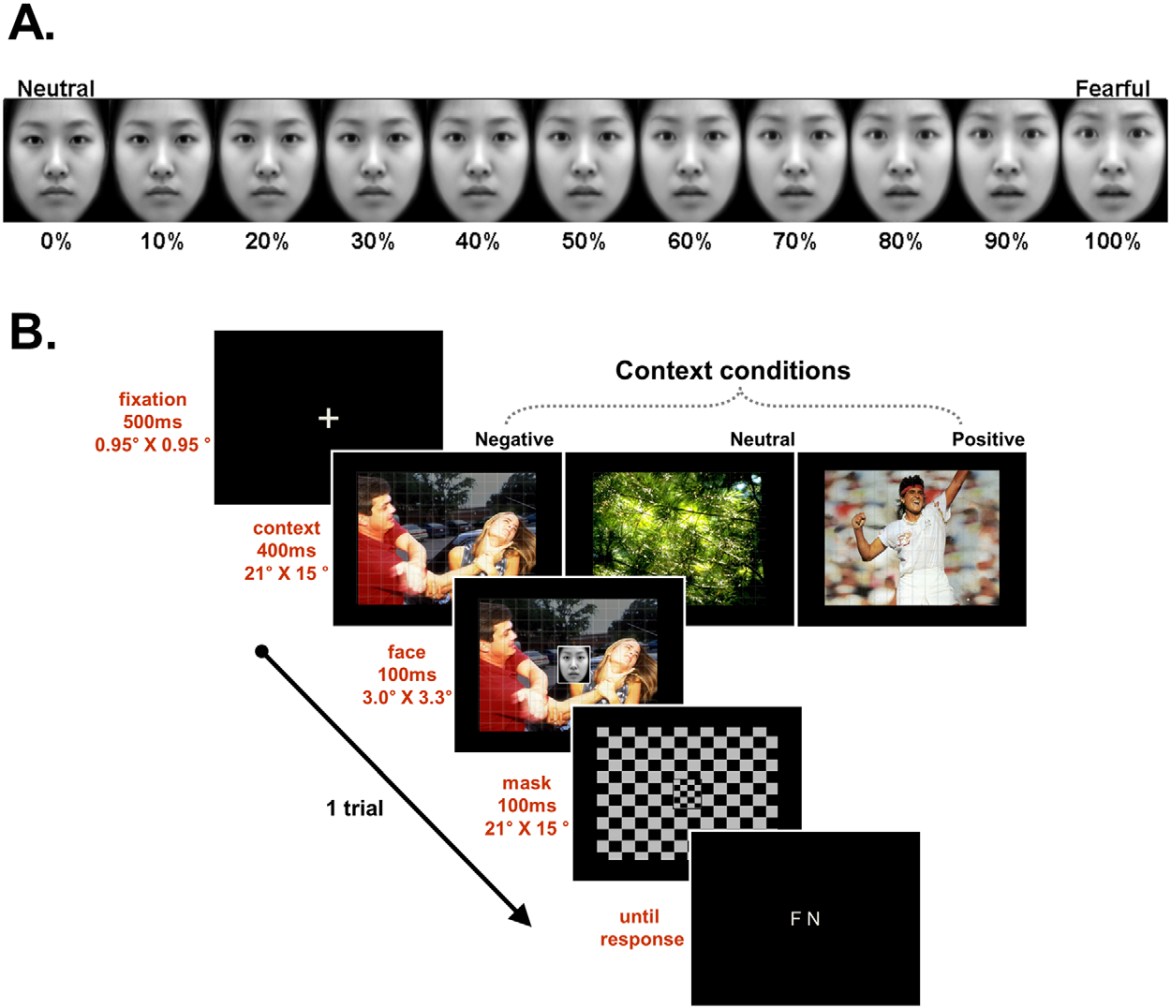
Experimental stimuli and design.

Face stimuli (fearful and neutral) were taken from the Korea University Facial Expression Collection [28]. To vary emotional intensity parametrically, we morphed the stimulus faces from neutral (0%) to fearful (100%) in 10% increments (**Figure 1A**). We employed 11 separate increments during the two-forced choice task, after equalizing the different face stimuli for luminosity. Each face was enclosed in a rectangular frame (3.0°×3.3°) excluding most of the hair and non-facial contours. We selected the context images from the International Affective Picture System (IAPS; [29]), employing 25 negative, 25 neutral, and 25 positive images (21°×15°) as negative, neutral, and positive context stimuli (see **Table. S1**). To obtain the observers’ unbiased responses, we used additional Fourier transformed images to create a baseline. Superlab 4.0 with a RB-730 response pad (Cedrus, CA) was used to program the experiment. Stimuli were presented on a personal computer's 21-in. display screen, at a viewing distance of approximately 60 cm.

### Procedure

Observers were told to place their index fingers on the response keys. The experiment consisted of one practice session of 10 trials, followed by four test sessions, one for each context: negative, neutral, positive, and baseline. Each session consisted of two blocks of 275-trials. Thus, each observer performed 2,250 trials (25-presentations of a face stimulus at each intensity×11 face intensities×2 blocks×4 contexts). Observers responded to the baseline condition first. Following the baseline session, the order of the other contexts was counterbalanced across observers. Rest breaks were given as necessary to avoid observer fatigue.

Each trial began with a 500 ms fixation cross (0.95°×0.95°), followed by 50 ms of blank screen. Observers were asked to stare at the fixation. Next, a context stimulus was presented first. After 400 ms, the stimulus face was presented centrally, on the context, for 100 ms. The context stimulus remained visible until the face stimulus terminated. At stimulus offset, a mask, which was a white-black checkerboard rectangle (21°×15°) replaced the stimuli. After 100 ms, the response screen appeared until the observer made a response (**Figure 1B**). Observers indicated whether the stimulus was a fearful or neutral face by pressing one of two response keys. The inter-trial interval was 1,000 ms.

### Curve fitting

To obtain psychometric curves for the different contexts, we fitted the behavioral data for each context to the curves via the Naka-Rushton contrast response model [30](see Equation 1)
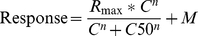
(1)


(Equation 1. Naka-Rushton Response Function)

–where *Response* represents the percentage of “fearful” responses, *C* is the stimulus intensity level (contrast), *C*50 is the contrast at half the saturating response (perceptual threshold), *n* is the exponent that determines the steepness of the function (slope), *R*_max_ is the maximum response relative to the baseline (asymptote), and *M* is the response at the lowest intensity level. To fit the data to the model, we constrained the parameters *C*50, *n*, and *R*_max_, based on each individual’s baseline context results. Curve fitting was performed with a maximum likelihood criterion. To best fit the data to the model, we fit the baseline condition data first with allowing each parameter (i.e. *C*50, *R_max_,* and *n*) to vary freely. Next, the curve fit to each context condition across observers was constrained fit bounds by first obtaining estimates from the baseline condition (final *C50* ranging from .2894 to .5863; *M* from 0 to .1168; *n* from .0572 to 14.84; *R*_max_ from .7269 to 1.0).

### Psychometric measures

To measure each observer’s information-processing characteristics, we used a BIS/BAS questionnaire that included one BIS scale and three BAS scales: Drive, Fun Seeking, and Reward. Because the BIS scale and the BAS reward scale depend upon one’s information processing tendencies toward external events, information, and the world [19], [31], we focused on BIS and BAS reward scales in the present study. To examine the relationship between BIS/BAS tendencies and contextual influences, context effect scores were calculated for both the negative and positive contexts, by subtracting the threshold value for the neutral context from that of the appropriate emotional context. A context effect score<0 indicated a decrease in the threshold.

## Results

### Context Modulation of Perceptual Threshold

**Figure 2:**
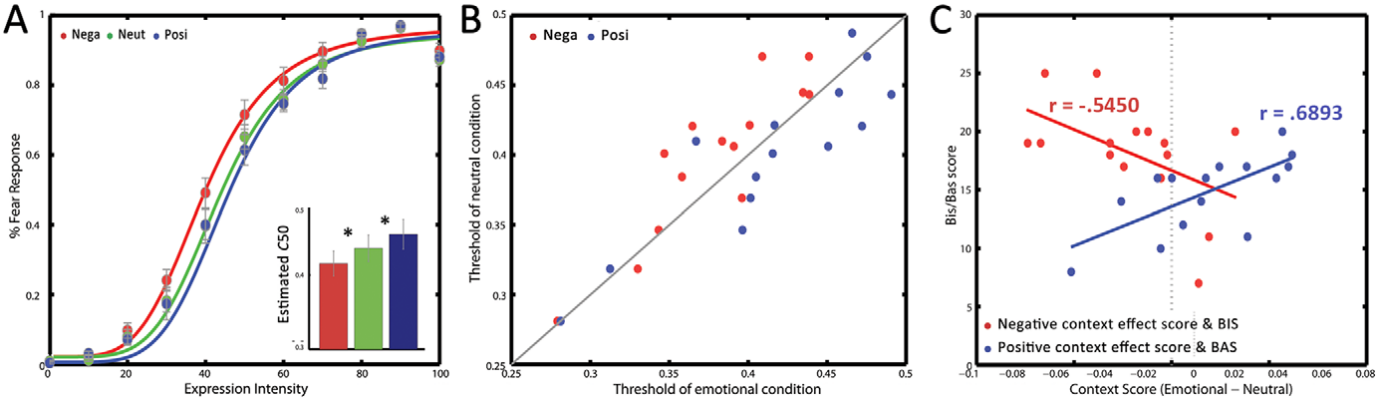
Results. (A) Psychometric functions fitted to the average response data. (B) The effects of emotional context on *C*50, for each observer. Points falling on the gray line represent unity, where there is no difference between emotional and neutral contexts. Points under the line indicate increased thresholds, and points over the line indicate decreased thresholds. (C) Scatter plot of the relationship between the observer’s context effect score and their informational tendency (BIS/BAS) with regard to facial emotion perception. A context effect score below zero indicates a decrease in the threshold value, and a context effect score above zero indicates an increase in the threshold value.

For average data, the estimated perceptual threshold (*C*50) function for fearful faces in a negative context shifted consistently to the left relative to its function in neutral context, when the expression intensity was ambiguous (30–60% intensities). The *C*50 function in the positive context shifted consistently to the right relative to that obtained in neutral context, even though modulation was not as great as it was in negative context (**Figure 2A and B**). Observers perceived more fearful faces in negative context (*C*50 = .3985) than in neutral context (*C*50 = .4312). Conversely, positive context led to a consistent increase in threshold (*C*50 = .4449) relative to neutral context.

To further explore the data, the individual psychometric functions were estimated (mean *R^2^* = .97, range  = .82–99). A statistical test revealed a significant difference in the *C*50 parameter between the context conditions, *F*(2, 28) = 7.83, *p*<.005, η^2^ = 36. A subsequent comparisons (*LSD*) showed that the threshold was significantly lower in negative context (C50 = .4154) than neutral context (C50 = .4360), *p*<.05, but it was marginally higher in positive context (C50 = .4548) than neutral context, *p*<.05. Also, there was a significant difference between negative and positive context, *p*<.01. The percentage of reported “fearful” responses in the intensity of the stimulus was not modulated by contextual information when expression intensity was low (0–20%) or high (70–100%), indicating that observers’ responses were based on the facial configuration rather than context image.

### Relationship between BIS/BAS and Context Effect

The individual’s information processing tendencies modulated the context effect obtained (**Figure 2C**). To examine the relative contributions of BIS and BAS tendencies to the context effect, a stepwise multiple regression analysis was conducted on the entire dataset, entering the BIS and BAS values as independent variables and the context effect score as the dependent variable. In the negative context, only BIS tendency contributed significantly to the context effect score (β = –.545, *p<*.05), whereas BAS tendency did not (β = −.055, *p = *.82). Conversely, in the positive context, BAS tendency contributed significantly to the score (β = .689, *p<*.005), while BIS did not (β = .367, *p = *.068).

## Discussion

Other people’s affective facial expression should be interpreted in terms of the context in which it occurs for people to adequately behave in social situation. The present study investigated how contextual information influences individuals’ facial emotion perception, by directly estimating each observers’ thresholds for perceiving facial expressions. The findings reported here support the idea that people generally tend to use contextual information when discerning the emotion of a face. The observer’s perceptual threshold (i.e. C50) for a fearful face changed as a function of contexts (i.e., shifting rightward), supporting the previous studies showing that context information could modulate facial emotion perception [6]–[14]. Surprisingly, individuals’ BIS/BAS levels correlated with the extent to which context information influenced facial emotion perception. When facial emotion was ambiguous, the negative contextual images strongly influenced the facial-expression perception of the observers with high BIS tendencies (*r* = –.545, *p<*.05), whereas positive images influenced the perception of the observers with high BAS tendencies (*r* = .689, *p<*.005).

Growing body of evidence indicates that facial emotion perception is modulated by the context. For example, Mobbs et al.,(2007) reported that both negative and positive contexts yielded a significant difference for face perceptions in behavioral rating and brain activities. Similarly, using a conditioning procedure, Lim and Pessoa (2008) have shown that the perceptual threshold was significantly lower for a face in a color background that has been previously paired with an aversive stimulus [32]. In comparison, our study, employing psychophysical measurement, revealed a bidirectional context modulation of face perception for fearful face (**Figure 2A**). The observers were more likely to perceive ambiguous faces (e.g., 30 – 60% intensities) as fearful in the negative context than they were in the neutral context. On the other hand, they were less likely to perceive the faces as fearful in the positive context. Taken together, these studies indicate that perceptual thresholds for emotional faces are modulated by functional relevance.

What are the mechanisms by which the context information modulated facial emotion perception? One possible explanation for such a shift in the C50 is that visual processing at the stage of facial encoding was modulated by contextual information. It has been known that the amygdala activity that emotional information triggers leads to enhanced visual processing [33](see also [34], ) through the neural connections between the visual area (e.g., V1) and the amygdaloid regions [36], [37]. Indeed, it has been found that contextual information leads to amygdala activation in face stimulus processing [9], [11]. For example, Mobbs et al. (2007) observed an interaction between the amygdala and the visual areas, including face processing regions such as fusiform gyrus (FFG) and superior temporal sulcus (STS), when they presented observers with faces in contextual scenes. The present findings regarding individual difference in BIS/BAS tendencies also support the above mechanism considering the previous researches showing relationships between amygdala activation and the level of BIS/BAS [23], [24]. That is, the level of BIS/BAS determines the degree of amygdala activation for the contextual emotional information, resulting in the degree of changes in the visual processing of the faces. Consequently, observers altered the perceived emotion from the face in accordance with their degree of sensitivity changes.

However, because amygdala activation is not just caused by negative information [38], [39], the positive context should have resulted in a decrease in the C50 function, too. The current findings-an increase in C50 function (i.e., a rightward shift) in the positive context-suggest that context information had an influence on the late-processing stages of interpretation and perceptual decisions, as well as at the early perceptual-processing stages. Indeed, Righart and de Gelder (2008b) found that observers’ responses to a facial expression they saw in an emotionally incongruent scene tended to be biased toward the contextual emotion, not to the face emotion, indicating that context information directly modifies observers’ interpretations of what a facial expression means in the late stages. Also, when perceiving the emotions of faces superimposed on emotional contexts, neural activation occurs in ventral prefrontal cortex (vPFC; [11]), which plays a key role in top-down processing, including inference, expectation, and decision-making [40]–[42]. That is, the present findings are consistent with the idea that the degree to which top-down processing contributes facial emotion perception depends on the observers’ subjective relevance or salience of emotional context information [32], [43], [44].

Although individual differences was found to be an important factor modulating such contextual influence on facial expression perception in the present study, other factors are worthy of consideration when researchers investigate contextual influences on face emotion perception. One such factor could be the observers' cultural differences. A recent study demonstrated that Japanese and American participants differed in utilizing contextual information for emotion perception [10]; the East Asians tended to perceive objects within their given context, whereas the Westerners focused more on each focal object, independently of its context [45]. Moreover, the effect of context on facial emotion perception also varies as a function of the observer’s age and level of stress [44].

As in previous studies [6]–[14], [44], the present results challenged the major view of facial expression perception indicating that facial configuration is interpreted in terms of its surrounding context information to perceive facial emotion, rather than it is just recognized in terms of its features. More important, the way how contextual information affects the facial expression perception depends on the observer’s individual tendency to process positive or negative information. These findings shed light on the factors that contribute to emotion perception for the face in the real world; perceptual cue, context information, and observer’s tendency to use context information. Thus, consideration of these factors is necessary to understand how to perceive emotion in a social setting.

## Supporting Information

Table S1 **Average Normative Valence and Arousal (Standard Deviations) and Picture Identification Numbers from the International Affective Picture System for the Negative, Neutral and Positive Pictures Used (valence scale ranging from 1: negative – 9: positive; arousal from 1: calm – 9: aroused).**
(DOCX)
