# Supplementary material for: Context Modulation of Facial Emotion Perception Differed by Individual Difference
Source: PLoS One. 2012 Mar 14;7(3):e32987. doi: 10.1371/journal.pone.0032987 (PMC3303876; doi:10.1371/journal.pone.0032987)
Supplement: Table S1 — Average Normative Valence and Arousal (Standard Deviations) and Picture Identification Numbers from the International Affective Picture System for the Negative, Neutral and Positive Pictures Used (valence scale ranging from 1: negative – 9: positive; arousal from 1: calm – 9: aroused). (DOCX) [file pone.0032987.s001.docx]

| **Negative** | **Neutral** | **Positive** |
| --- | --- | --- |
| Valence = 2.90 (.55) | Valence = 5.58 (.97) | Valence = 7.47 (.54) |
| Arousal = 6.77 (.53) | Arousal = 3.31 (.75) | Arousal = 5.73 (.96) |
| 1120 | 1590 | 1340 |
| 1300 | 1602 | 1440 |
| 2683 | 1620 | 1710 |
| 3071 | 1670 | 1999 |
| 3102 | 2191 | 2040 |
| 3120 | 2235 | 2070 |
| 3130 | 2480 | 2091 |
| 3550 | 2514 | 2208 |
| 6212 | 2579 | 2224 |
| 6315 | 2590 | 2340 |
| 6540 | 5250 | 2341 |
| 6550 | 5390 | 2345 |
| 6560 | 5530 | 2501 |
| 9040 | 5593 | 2530 |
| 9181 | 5750 | 2550 |
| 9230 | 7140 | 4598 |
| 9253 | 7170 | 4599 |
| 9265 | 7175 | 4623 |
| 9400 | 7200 | 4626 |
| 9410 | 7211 | 8320 |
| 9433 | 7233 | 8330 |
| 9570 | 7491 | 8350 |
| 9611 | 7500 | 8380 |
| 9910 | 7510 | 8461 |
| 9921 | 7950 | 8470 |
